# Alkylammonium Spacer-Directed Charge-Transfer in 2D|3D Perovskite Solar Cells

**DOI:** 10.1021/acsomega.5c10453

**Published:** 2026-01-28

**Authors:** Barbara Scola Rodrigues, Lucas Polimante, Cleyton Alexandre Biffe, Carlos Alberto Rodrigues Costa, André Sarto Polo

**Affiliations:** † Centro de Ciências Naturais e Humanas, 425753 Universidade Federal do ABC, 09210-580 Santo Andre, SP, Brazil; ‡ Brazilian Nanotechnology National Laboratory (LNNano), Brazilian Center for Research in Energy and Materials (CNPEM), 13083-970 Campinas, Sao Paulo, Brazil

## Abstract

Long-term stability is a key challenge for Perovskite Solar Cells (PSCs). A promising strategy is to construct two-dimensional|three-dimensional (2D|3D) lead-halide perovskite heterostructures, which integrate the high efficiency of 3D phases with the high durability of 2D layers. These layers are constructed on 3D perovskites by spreading spacer solutions onto their surfaces, forming 2D|3D structures. Here, we systematically investigate the role of two alkylammonium spacers by using butylammonium iodide (BAI, a Ruddlesden–Popper spacer) and butyl-1,4-diammonium diiodide (BDAI_2_, a Dion-Jacobson spacer) in two different concentration regimes. At low spacer concentrations (5 mmol L^–1^ BAI and 0.5 mmol L^–1^ BDAI_2_), both spacers primarily acted as surface passivators, yielding efficiencies comparable to those of pristine methylammonium lead iodide perovskite. High spacer concentrations (50 mmol L^–1^ BAI and 5 mmol L^–1^ BDAI_2_) induced layered 2D|3D phases with contrasting effects. BAI promoted structural flexibility (by varying *n* from 1 to 2) and improved moisture resistance, thereby enhancing device stability. BDAI_2_ unexpectedly leads to rapid degradation under ambient processing conditions. Photoluminescence, conductive atomic force microscopy, and electrochemical impedance spectroscopy confirmed that excessive spacer incorporation introduces insulating barriers that hinder charge transport. Optimized concentrations suppress nonradiative recombination without compromising conductivity. Durability tests demonstrated that BAI consistently prolonged device lifetime, while BDAI_2_ devices degraded rapidly. These results reveal that spacer chemistry and concentration critically determine the trade-off between charge transport and environmental resilience in 2D|3D PSCs. By clarifying these mechanisms, this work establishes insights into the rational design of spacer-assisted perovskites, offering a pathway toward more durable and commercially viable perovskite photovoltaics.

## Graphical abstract

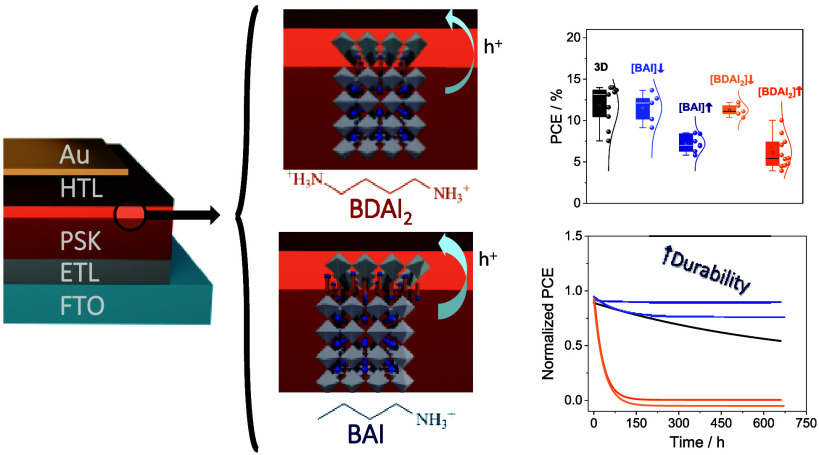

## Introduction

Organic–inorganic metal halide perovskite solar cells (PSCs) have been widely reported in the literature as an emerging photovoltaic (PV) technology, offering a cost-effective alternative to silicon solar cells.^1,2^ Since their initial development, PSCs have demonstrated rapid improvements in power conversion efficiency (PCE), approaching the Shockley–Queisser theoretical limit.^3^ The most studied PSCs compositions are based on methylammonium (MA^+^, CH_3_NH_3_^+^) lead halide, such as CH_3_NH_3_PbI_3_.^4^ CH_3_NH_3_PbI_3_-based perovskites exhibit exceptional optoelectronic properties, a broad overlap with the solar spectrum, low trap-state densities, and low exciton binding energies (≈10–50 meV).^5,6^

Despite the advancements these devices have achieved, some challenges remain to be addressed for the technology to be commercially viable, with the main issue being long-term stability.^7^ Factors such as moisture,^8,9^ heat,^10^ and light exposure^11^ induce perovskite decomposition,^12^ reducing the efficiency of the devices and limiting their commercial viability.^13^ One strategy to mitigate this issue is the use of two-dimensional|three-dimensional (2D|3D) heterointerfaces, where the bulk 3D layer is responsible for the outstanding performance of the device, while the resistant 2D capping layer is formed on top of the 3D perovskite. In this way, it is possible to join the good performance of the 3D film with the high durability of the 2D one, resulting in highly efficient and durable devices.^14^

In a typical 3D perovskite, the crystal structure consists of a network of corner-sharing [PbI_6_]^4–^ octahedra, with small organic cations occupying the interstitial spaces (Figure 1a). To form 2D perovskites on top of this structure, bigger organic cations (spacers) replace the organic cation present in the 3D structure, physically separating the [PbI_6_]^4–^ layers. The number of inorganic octahedral layers between these organic spacers is denoted by *n*. For *n* = 1, the structure comprises a single octahedral layer flanked by organic cations (Figure 1b,c), and for *n* = 2, two octahedral layers are present (Figure 1d). As *n* increases, the perovskite slabs become thicker, gradually approaching the electronic and structural characteristics of 3D perovskites (Figure 1e,f).^15^ Using spacers solutions onto 3D perovskite surface, it is possible to prepare the 2D|3D structure. The hydrophobic feature of the spacer enhances moisture resistance, improving the durability of the device.^16^

**Figure 1:**
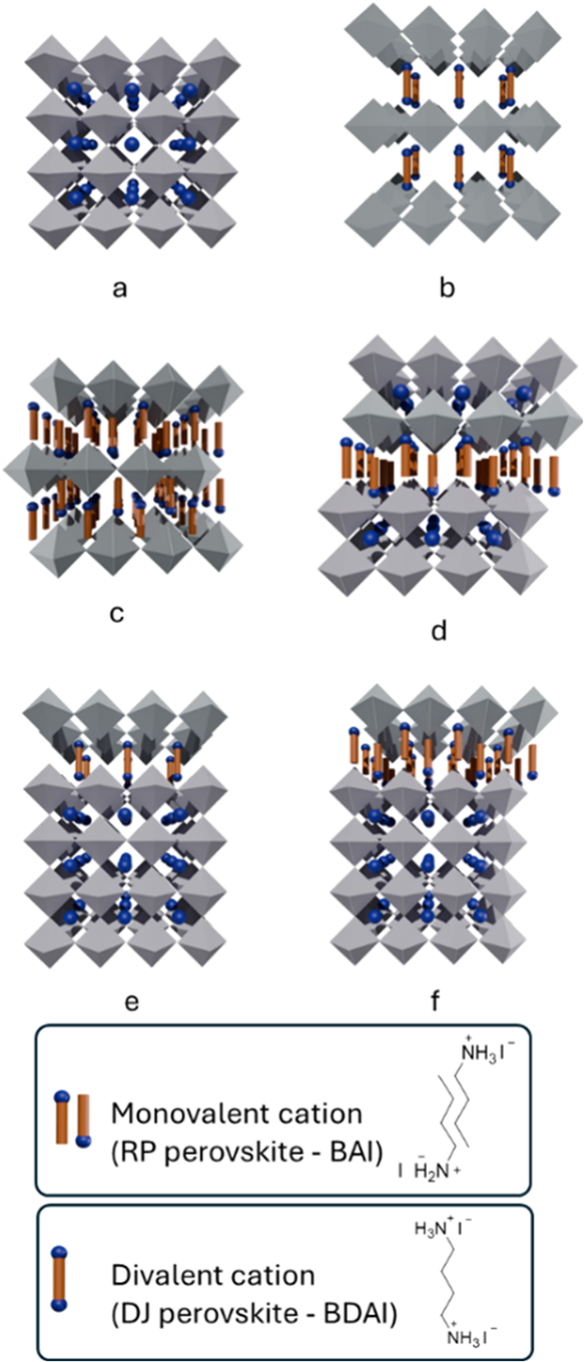
Structural representations of 3D perovskite (a), 2D perovskites with Dion–Jacobson (divalent spacer, *n* = 1) (b, c) phases, 2D perovskite (RP) with *n* = 2 (d), and mixed 2D|3D perovskites DJ (e) or RP (f).

Besides their durability, several authors mention that 2D perovskites can passivate surface traps, thereby reducing defect recombination in devices using 2D|3D films,^17^ and the Ruddlesden–Popper (RP) and Dion–Jacobson (DJ) phases have gained significant interest due to their structural diversity and potential impact on PSC performance.^18–21^ RP perovskite is composed of monoammonium spacer cations that rely on van der Waals forces between cations to bind perovskite slabs, and DJ perovskites employ diammonium cations that can connect both inorganic layers through ionic bonds (Figure 1). The RP phase is commonly associated with perovskite structural instability due to its weak interlayer bond; in contrast, the DJ phase provides rigidity to the perovskite framework.^22,23^ Even though rigidity provides a higher degree of structural stability, it still limits tolerance to lattice distortions that can hinder charge carrier transport.^22,24,25^

Another key challenge to perovskite stability is cationic migration, particularly with aliphatic 2D spacers.^26^ Although some authors suggest that the introduction of spacer cations mitigates ionic migration, recent studies have highlighted their influence on ion transport and phase segregation.^23,24^ In traditional 3D perovskites, halide ion migration is a well-known degradation pathway, but in 2D|3D systems, the mobility of large organic spacers and their interactions with the inorganic framework can also contribute to instability. Specifically, aliphatic diammonium spacers in DJ-type perovskites introduce stronger electrostatic interactions, potentially reducing ion migration.^22,27^ However, the restricted octahedral movement in DJ structures may also induce internal strain, affecting charge carrier transport and long-term device performance.^28^

Many papers have reported the role of spacer concentrations in 2D|3D PSCs,^23,26,29,30^ mostly focusing either on their impact on surface passivation or durability improvements. However, these investigations did not systematically compare the relationship between concentration and properties.^23,29^ Furthermore, although both RP and DJ 2D perovskites have been widely explored, few studies directly compare their behavior across different spacer concentration regimes.^31–33^ The relationship of spacer concentration, phase formation, and defect tolerance remains barely understood, particularly in the context of how these factors influence the trade-offs between structural stability and charge-carrier transport.

In this work, we systematically investigated two types of spacers, butylammonium iodide (BAI) as an RP spacer and 1,4-Butanediammonium diiodide (BDAI_2_) as a DJ one. These spacers were chosen because they have the same number of carbons on the aliphatic chain aiming to avoid misunderstandings due to the difference in this parameter. The properties of the obtained 2D|3D perovskite were evaluated in distinct concentrations to ensure the formation of *a* well-defined 2D phase on the surface of the 3D perovskite or, in lower concentrations, the spacer acts as *a* passivating agent rather than forming *a* complete layered structure. Using this approach, we aim to provide *a* new perspective on how structural rigidity, charge transport, and defect passivation interplay in multidimensional perovskites; not only offering *a* deeper understanding of the fundamental mechanisms governing perovskite stability and efficiency but also providing valuable design strategies for optimizing PSC performance by balancing the advantages of 2D structural protection with the electronic benefits of *a* predominantly 3D perovskite phase.

## Materials and Methods

### Materials

Titanium di-isopropoxide bis­(acetylacetonate), 1-butanol ≥99.4%, titania paste (transparent, 20 nm average particle size), Ethanol ≥99.5%, methylammonium iodide 98%, *N*,*N*-dimethylformamide (DMF) 99.8%, dimethyl sulfoxide (DMSO) ≥99.9%, chlorobenzene 99.8%, acetonitrile ≥99.9%, lithium bis­(trifluoromethylsulfonyl)­imide (LiTFSI) 99.9%, 2,2′,7,7′-tetrakis­(*N*,*N*-di-*p*-methoxyphenylamine)-9,9′-spirobifluorene (Spiro-OMeTAD) 99% HPLC, tris­(2-(1*H*-pyrazol-1-yl)­pyridine)­cobalt­(II) di­[bis­(trifluoromethane)­sulfonimide] (FK102 Co­(II) TFSI) 98%, 4-*tert*-butylpyridine 96%, and 2.2 cm glass coated with FTO (8 Ω/sq), ethyl acetate 99.8%, butylamine 99.5% (BA), 1,4-diaminobutane 99% (BDA), hydriodic acid 57 wt % (HI), 2-propanol 99.5% (IPA), were purchased from Sigma-Aldrich. All solvents used in preparations are anhydrous. Lead­(II) Iodide 99.99% was purchased from TCI chemicals. All materials were used without further purification.

### 2D Spacer Preparation

The molecular precursors, butylamine (BA) and butane-1,4-diamine (BDA), were dissolved in 10 mL of anhydrous ethanol in a vial and stirred in an ice bath. Hydroiodic acid (HI, 57 wt % in water) was then added dropwise to the solution, maintaining molar ratios of BA/HI = 1:1.1 and BDA/HI = 1:2.2. The resulting solution was stirred for 2 h to ensure complete reaction. The resulting solution was concentrated by rotary evaporation (50 °C) and the crude product was washed with diethyl ether, collected by vacuum filtration, and dried in a vacuum oven at 60 °C for 24 h to ensure complete solvent removal.

### Solutions’ Preparation

The compact TiO_2_ (c-TiO_2_) precursor solution was prepared by diluting 90 μL of titanium diisopropoxide bis­(acetylacetonate) in 1 mL of 1-butanol. The solution was stirred immediately before its use for spin-coating the compact electron transport layer. Preparation and deposition were conducted under ambient conditions.

The mesoporous TiO_2_ (m-TiO_2_) suspension was prepared by diluting 0.16 g of the commercial titania paste in 1 mL of absolute ethanol. The freshly prepared mixture was vigorously stirred and then briefly sonicated to ensure uniform dispersion before spin coating.

The perovskite precursor solution was prepared by dissolving 1.25 mol L^–1^ of PbI_2_ and 1.25 mol L^–1^ of methylammonium iodide (MAI) in a 4:1 volume mixture of DMF/DMSO.^34^ The solution was stirred for 2 h at room temperature in a sealed vial to be fully dissolved and homogeneous. It was stored under a dry, inert atmosphere until deposition.

The 2D spacer solutions were prepared by dissolving either butylammonium iodide (BAI) or butane-1,4-diammonium diiodide (BDAI_2_) in IPA. BAI was dissolved at concentrations of 50 mmol L^–1^ or 5 mmol L^–1^, while BDAI_2_ was prepared at 5 mmol L^–1^ or 0.5 mmol L^–1^. All solutions were stirred by ultrasonication for 2 h to ensure its complete homogenization.

The hole transport layer (HTL) solution was prepared by dissolving 0.07 g of Spiro-OMeTAD in 1 mL of chlorobenzene. To this solution, 17.5 μL of a LiTFSI stock solution (1.8 mol L^–1^ in acetonitrile) and 15.4 μL of FK102 Co­(II) TFSI (0.28 mol L^–1^ in acetonitrile) were added as a dopant. The mixture was stirred until homogeneous and then filtered before being spin-coated. The doped Spiro-OMeTAD solution was stored in the dark under dry conditions and used within a few hours of preparation.

All the preparation of the solutions and deposition steps were performed in ambient conditions with relative humidity (RH) 40–50%.

### Device Fabrication

Device fabrication was carried out with slight modifications, mainly in the perovskite layer deposition, on the procedures previously reported.^34–37^ Before deposition, 2 cm × 2 cm FTO glasses were cleaned sequentially via ultrasonication for 20 min in a 20% solution of Extran, then in water, acetone, and finally, isopropyl alcohol. The c-TiO_2_ layer was deposited by spin coating 150 μL of the titanium diisopropoxide bis­(acetylacetonate) solution using a three-step spin program: 700 rpm for 12 s, 1000 rpm for 10 s, and 2000 rpm for 30 s. The films were then heated at 125 °C for 10 min and then treated with ozone for 30 min. Onto this film, 150 μL of a TiO_2_ paste suspension in ethanol was spin-coated at 2000 rpm for 30 s. The resulting m-TiO_2_ films were dried at 100 °C for 5 min and then annealed at 550 °C for 1 h. After cooling, the substrates underwent a final 30 min ozone treatment before perovskite deposition.

The perovskite layer was deposited by spin coating 150 μL of the perovskite precursor solution at 2000 rpm for 25 s. After 15 s of spinning, 300 μL of ethyl acetate was dynamically dropped onto the spinning substrate. The films were then annealed at 100 °C for 10 min. The 2D perovskites were prepared by spin coating the spacer solutions (BAI or BDAI_2_) at 5000 rpm for 30 s onto the perovskite films using the concentration described before (solutions preparation section). The films were dried at room temperature or thermally treated.

The HTL was deposited by spin coating 150 μL of its solution at 5000 rpm for 30 s. Then, gold counter-electrodes were thermally evaporated, as described elsewhere,^35^ forming a gold film with a thickness of 60 nm. Each substrate results in four devices, with an electrode area of 20 mm^2^ each.

### Characterization

The photovoltaic performances of the fabricated PSCs were evaluated under irradiation provided by a solar simulator (Newport, model 96000) equipped with an AM 1.5G filter, operating at 1 sun (100 mW cm^–2^), and a source-meter (Keithley, model 2410) applied the voltage and measured the corresponding current, resulting in the current density–voltage (*JV*) curves. The active area was defined by masking the solar cells to an exposure size of 0.07 cm^2^. The photovoltaic parameters (*V*_OC_, *J*_SC_, FF, and PCE) were determined from the *JV* curves. Durability tests were performed following the ISOS-D1 protocol (shelf life),^38^ where the solar cells are stored in the dark and ambient air. The reported photovoltaic parameters represent the average values (*V*_OC_, *J*_SC_, FF, and PCE) obtained from three devices per composition.

External Quantum Efficiency spectra were measured by a system that consists of a 300 W Xe lamp (Newport - model 6258) placed in a lamp housing that illuminates the entrance slit of a 0.25 m Czerny–Turner monochromator (Newport - Cornerstone 260), equipped with a diffraction grating of 1200 lines mm^–1^ and appropriate band-pass filters to avoid second-order effects. The quasi-monochromatic light beam exits the monochromator and the light intensity is measured at each wavelength by a silicon detector, (Newport - model 818-UV), which was connected to a virtual power-meter, (Newport model 841-p-USB). The photocurrent was measured by placing the solar cells at the same position of the silicon detector, and measuring the photocurrent generated at each wavelength by using a source-meter (Keithley model 2410). The EQE value was determined mathematically by using the wavelength, incident light intensity and photocurrent density generated as described elsewhere.^39^ The integrated photocurrent densities derived from the EQE spectra were compared with the *J*–*V*-derived *J*_SC_ values to ensure internal consistency.

Absorption spectra were recorded using an Agilent 8454 diode-array spectrophotometer. For the photoluminescence (PL) measurements a PicoQuant Fluotime 300 fluorescence spectrometer was employed, using a LED as light source (λ_exc_ = 635 nm). Scanning electron microscopy (SEM) images were collected by using a JSM 6701F (JEOL) field emission scanning electron microscope.

Grazing-incidence X-ray diffraction (GI-XRD) measurements were performed using a D8 Discover X-ray diffractometer (Bruker AXS) with Cu radiation source to investigate the near-surface crystallographic structure of the perovskite films. The incident angle (ω) was fixed at 1.0° to enhance surface sensitivity and minimize the substrate contribution. 2θ ranging up to 15°, and frame acquisition time of 300 s per scan. X-ray diffraction (XRD) patterns were obtained using a D8-FOCUS XRD (Bruker AXS) with a Cu Kα radiation source operating at 40 kV and 40 mA. The XRD profiles were collected between 4°< 2θ < 20°.

Conductive Atomic Force Microscopy (c-AFM) measurements were performed using a NX-10 (ParkSystems) with a PtIr-coated silicon probe model PPP-EFM (NanoSensors) in contact mode. A negative bias was applied to the tip and the current limited to a maximum of 5 nA. The scans were acquired over a 1.5 μm × 1.5 μm area (@256 × 256 pixels), in a controlled environment (<5% relative humidity). Electrochemical Impedance Spectroscopy (EIS) data were measured using a Metrohm Autolab (PGSTAT 204 equipped with a Frequency Range Analyzer FRA32M), with an alternating current perturbation potential (*V*_AC_) of 10 mV, at *V*_OC_ and frequencies ranging from 1 MHz to 0.1 Hz. The measurements were conducted under simulated sunlight irradiation (1 sun, 100 mW cm^–2^; AM 1.5G) provided by a solar simulator (Asahi Spectra, model HAL-320). The Nyquist diagrams were fitted using ZView software (v4.0d).

## Results and Discussions

To fabricate 2D|3D perovskite, we employed the sequential deposition method, in which a solution containing spacer cations is deposited onto *a* formed 3D perovskite film. This approach has been widely adopted to promote layered growth at the surface, enabling the formation of well-defined 2D|3D interfaces and resulting in devices with good performance.^40,41^ Previous reports have shown that low concentrations (1–5 mmol L^–1^) of monovalent (such as butylammonium iodide-BAI) or divalent (Butyl 1,4-diammonium diiodide-BDAI_2_) organic cations are enough to induce 2D features such as excitonic absorption bands, layered diffraction patterns, and enhanced surface passivation onto 3D perovskite surface.^42,43^ Based on these reports, our initial experiments reproduced these conditions by modifying the 3D perovskite with 5 mmol L^–1^ of BAI or BDAI_2_. Additional concentrations were subsequently examined to explore how spacer loading influences the emergence of 2D-layer signatures.

We initially characterized the samples by using UV–vis absorption spectra of perovskite films modified with different concentrations of 2D spacer cations. This technique reveals a dependence of characteristic features of both 3D and 2D perovskite phases on the concentration used, Figure 2. The 3D perovskite exhibits a broad absorption onset around 730 nm corresponding to the CH_3_NH_3_PbI_3_ bandgap, while additional peaks near 500 nm are attributed to excitonic transitions of the low-dimensional (2D) perovskite structures. As expected, a 3D band was present for all samples. For BAI-modified samples at 50 mmol L^–1^ (Figure 2a), it was possible to notice a 2D band at 501 nm, assigned to the phase *n* = 1. In the case of BDAI_2_ (Figure 2b), the film prepared from a 5 mmol L^–1^ solution also exhibits an additional absorption feature in the same region. However, it shows a broad excitonic feature that likely originates from a distribution of low-dimensional domains or structural inhomogeneity, rather than from a single well-defined *n*-phase.^44–46^ Such broadened bands are reported for DJ-type perovskites, where mixed-n stacking, incomplete layer ordering, or local disorder smear out the characteristic excitonic transitions.^22,44,47^ It is important to note that spectrophotometry is not the most suitable technique for identifying surface modifications. However, if the characteristic 2D peak is observed, it can be ensured that a 2D layer is formed.

**Figure 2:**
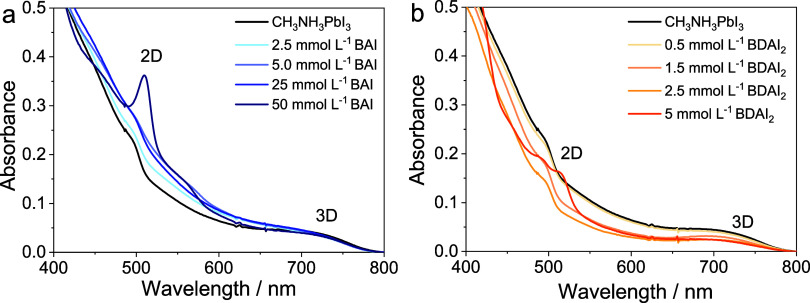
Absorbance spectra of 2D|3D perovskite films prepared using different concentrations of solutions of BAI (a) or BDAI_2_ (b).

To systematically evaluate the influence of spacer chemistry and concentration, two concentration regimes were selected for each spacer (BAI or BDAI_2_). These values were established based on both the chemical valency of the ammonium cations and the evolution of excitonic features observed in the UV–vis spectra, which clearly differentiate the passivation and 2D-layer formation regimes. It is worth mentioning that BDAI_2_ is not soluble in concentrations higher than 5 mmol L^–1^, therefore, this was chosen as the upper limit concentration, as well.

Structural characterization of pristine and spacer-modified CH_3_NH_3_PbI_3_ films was performed using grazing incidence X-ray diffraction (GI-XRD, Figure 3a–e) and conventional Bragg–Brentano geometry (XRD, Figure 3f) to investigate the formation of 2D phases. GI-XRD provides surface-sensitive information on crystalline phases and near-surface order. The pristine CH_3_NH_3_PbI_3_ film (Figure 3a) shows strong reflections around 14.2° corresponding to the tetragonal perovskite phase, along with a weaker reflection near 12.8°, due to residual PbI_2_, which is consistently present in all GI-XRD patterns.

**Figure 3:**
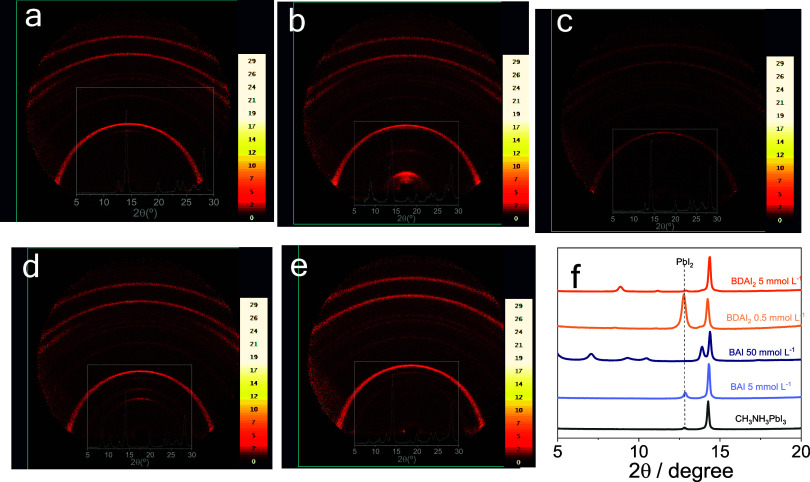
Grazing-incidence X-ray diffraction (GI- XRD, incidence angle = 1°) patterns of pristine 3D CH_3_NH_3_PbI_3_ (a), 3D CH_3_NH_3_PbI_3_ treated with [BAI] = 50 mmol L^–1^ (b), [BAI] = 5 mmol L^–1^ BAI (c), [BDAI_2_] = 5 mmol L^–1^ (d), or [BDAI_2_] = 0.5 mmol L^–1^ (e). Conventional Bragg–Brentano geometry XRD pattern of the samples (f).

Comparing the images of the 2D|3D samples prepared, it can be observed that using 50 mmol L^–1^ of BAI (Figure 3b), low-angle reflections clearly emerge near 4.5°, corresponding to a large interlayer spacing typical of the *n* = 1 Ruddlesden–Popper phase. This reflection is generally indexed as a 00l lamellar spacing, consistent with previously reported RP structures.^20,48,49^ A weak diffraction feature is also observed between ∼6–8°, a region commonly associated with overlapping higher-order (00l) reflections or higher-n RP phases (*n* ≥ 2). At 5 mmol L^–1^ BAI (Figure 3c), the low-angle signal is not observed, consistent with a passivation-dominated regime and minimal 2D phase development.

BDAI_2_-modified films prepared with 5 mmol L^–1^ solution (Figure 3d) exhibit broad diffraction features at 2θ = 8.7°, ascribed to the (00l) out-of-plane stacking reflection of a Dion–Jacobson (DJ) *n* = 1 phase.^27,50^ An additional broad signal appears near 10°, suggesting the coexistence of mixed-n DJ phases or disordered stacking arrangements.^44^ This observation points to a structurally heterogeneous or partially ordered layered configuration. The formation of well-defined higher-*n* DJ phases (*n* > 1) is rarely reported, as the rigid diammonium spacer constrains the interlayer separation and limits structural relaxation.^22^ This constraint introduces lattice strain that promotes local distortions and nonuniform stacking, favoring structural inhomogeneity over coherent higher-n configurations. At lower spacer concentration BDAI_2_ 0.5 mmol L^–1^, the same diffraction features are present but with significantly lower intensity, consistent with reduced 2D phase content.

Overall, the GI-XRD analysis reveals that the degree of phase uniformity is strongly influenced by spacer chemistry: BAI leads to more uniform and well-oriented phases, whereas BDAI_2_ produces a less ordered layered arrangement. These observations are consistent with other reports on mixed-dimensional perovskites.^51,52^

These surface-sensitive observations are reinforced by the conventional XRD patterns (ICSD 241482 - Figure 3f), where the main perovskite (110) peak (14.2°) and PbI_2_ (12.8°) signals are again observed. The agreement between GI-XRD and XRD confirms that both surface and bulk regions are influenced by spacer chemistry and concentration. The observation of 2D-related peaks at high spacer loading, particularly with BAI, highlights the spacer-dependent dimensional tuning and its effect on crystallographic structure.^50^

The presence of the 2D phase should increase the long-term stability of these films. To assess the durability of the films prepared, UV–vis spectra of the RP and DJ samples (BAI 50 mmol L^–1^ and BDAI_2_ 5 mmol L^–1^) were collected over several days (Figure 4). Both films initially displayed an absorption peak around 500 nm associated with low-dimensional layered domains. In the BAI-modified sample, this band associated with *n* = 1 gradually decreased, revealing a new peak at 517 nm, due to the formation of *n* = 2 phase (Figure 4a,c). Similar features have already been reported in the literature for aliphatic cations.^53^

**Figure 4:**
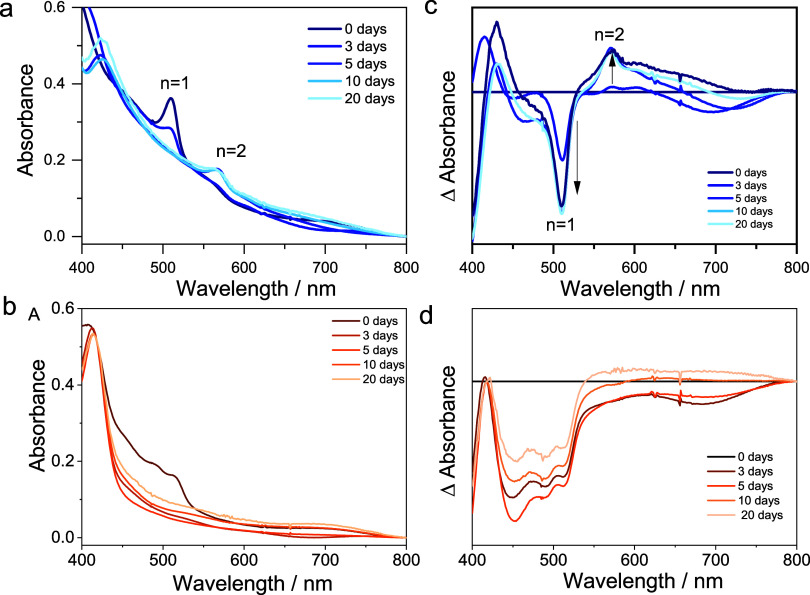
UV–vis absorption spectral changes of 2D|3D samples prepared by using [BAI] = 50 mmol L^–1^(a) or [BDAI_2_] = 5 mmol L ^–1^ (b) and their respective differential absorption spectra (c, d) for freshly prepared samples, and after 3, 5, 10, and 20 days.

In contrast, the BDAI_2_-modified sample (5 mmol L^–1^, Figure 4d), as discussed previously (Figure 2b) does not display a defined absorption peak (*n* = 1). Instead, it shows a broad and nonstructured absorption feature in the same spectral region, which gradually decreases over time.^22,46^ This behavior differs fundamentally from the BAI-treated film, which evolves from *n* = 1 to *n* = 2 and indicates that the DJ-derived layered domains do not exhibit a similar phase transformation during storage.

The absence of emerging *n* > 1 features over time supports the notion that BDAI_2_ suppresses phase evolution, as reported in the literature.^53,54^ The gradual loss of the 2D feature over time likely reflects migration or redistribution of BDAI_2_ cations into the 3D bulk, where they may act as passivating agents rather than promoting the formation of additional 2D layers.

This degree of phase instability is unexpected for DJ-type perovskites, whose strong interlayer connectivity is typically associated with enhanced structural robustness. However, their stability is extremely sensitive to how the films are processed: nonideal crystallization pathways, humidity during deposition, and rapid solvent removal can trap metastable or inhomogeneous 2D domains, which degrade much more rapidly.^55,56^

Absorption spectra of 3D perovskite and 2D|3D perovskites modified with 5 mmol L^–1^ BAI and 0.5 mmol L^–1^ BDAI_2_ were also monitored over time (Figures S7–S9). As shown in Figure 2, these samples only exhibited a band at ∼730 nm, with no evidence of 2D phase formation due to the lower spacer concentration. Pristine 3D perovskite displayed clear signs of degradation, evidenced by the progressive decrease in its absorption intensity. In contrast, the spectra of the BAI- and BDAI_2_-modified films remained relatively unchanged, likely due to the reduced 2D content or a slight passivation effect that mitigates degradation.

Steady-state photoluminescence (PL) was employed to investigate the electronic properties of films modified with spacer cations under excitation at 635 nm (Figure 5). The pure 3D perovskite exhibited a PL peak around 760 nm. Upon the modification with 50 mmol L^–1^ BAI or 5 mmol L^–1^ BDAI_2_, a significant decrease in PL intensity was observed, which can be attributed to the increasing coverage of 2D perovskite on the 3D perovskite surface. These 2D domains promote the redistribution or transfer of part of the photogenerated carriers across the 2D|3D interface and avoiding the electron–hole recombination responsible for the photoluminescence. Thus, reducing the recombination phenomenon, the radiative emission signal is weaker.^57^ A similar trend, in a lesser extent, was observed for the sample containing 5 mmol L^–1^ BAI. However, when 0.5 mmol L^–1^ BDAI_2_ was used, the emission intensity increased, likely due to surface defect passivation, which reduces nonradiative recombination processes.^28,58^

**Figure 5:**
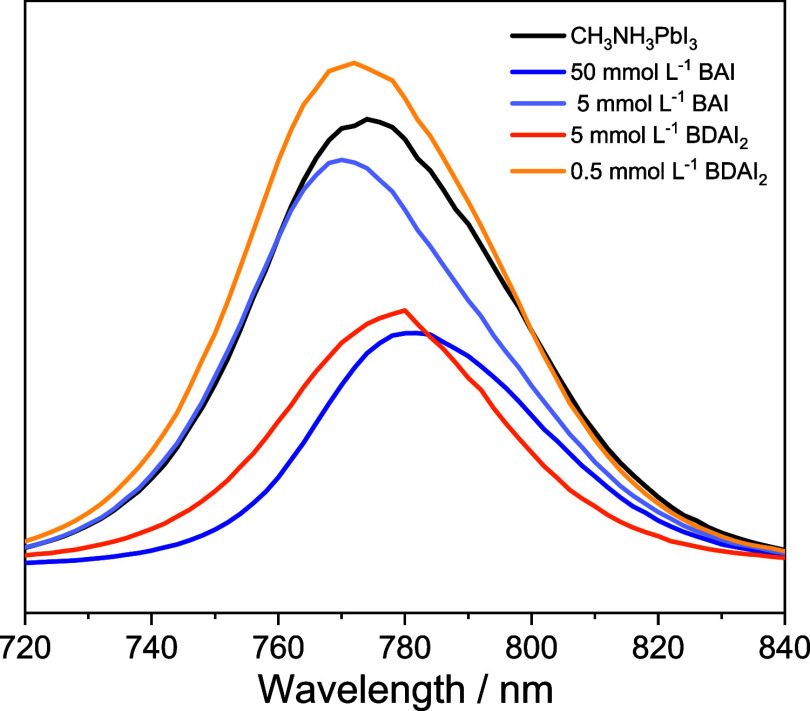
Steady-state photoluminescence spectra of 3D and 2D|3D perovskites incorporating BAI (50 or 5 mmol L^–1^) or BDAI_2_ (5 or 0.5 mmol L^–1^). (λ_exc_ = 635 nm).

To further investigate the influence of incorporating BAI or BDAI_2_ spacers on local charge transport, conductive atomic force microscopy (c-AFM) was performed on the 3D or 2D|3D perovskite films. Figure 6 presents the topography (top) and corresponding current maps (bottom) for pristine CH_3_NH_3_PbI_3_ and those prepared using [BAI] = 5 mmol L^–1^, [BAI] = 50 mmol L^–1^, [BDAI_2_] = 0.5 mmol L^–1^ or [BDAI_2_] = 5 mmol L^–1^. The topography and current maps revealed strong correlations between spacer concentration, surface characteristics, and nanoscale conductivity.

**Figure 6:**
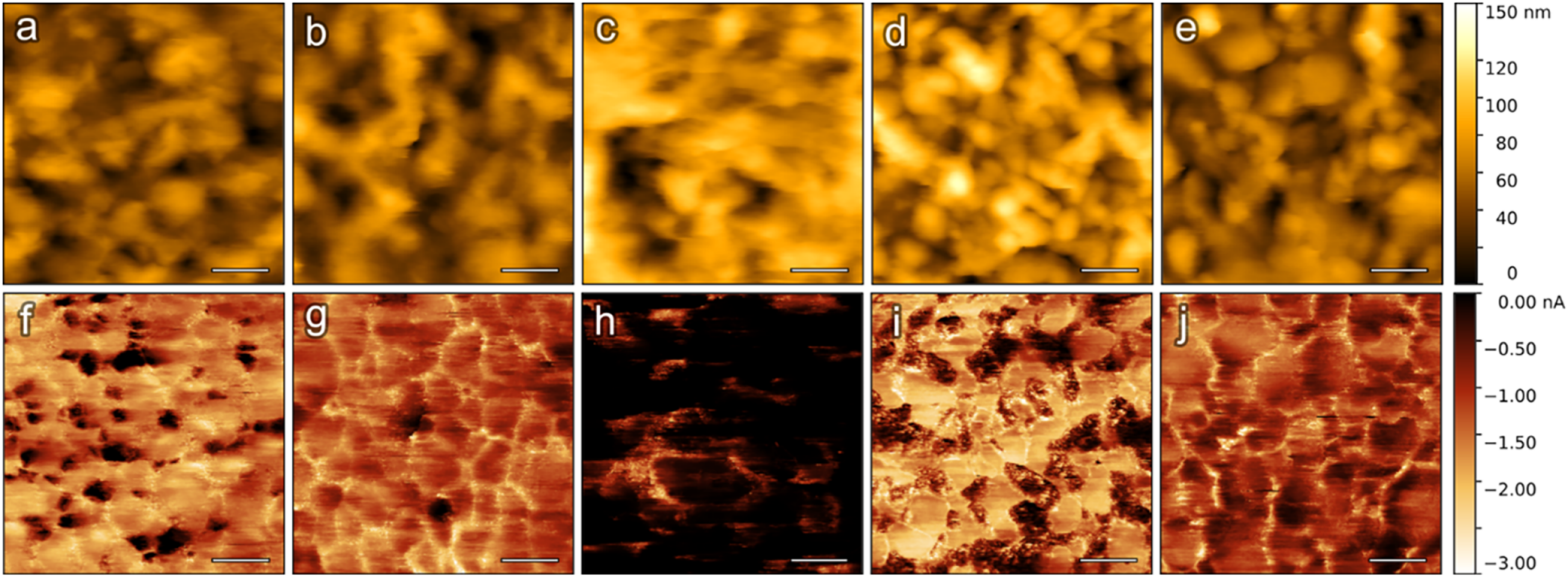
Topography (top) and corresponding c-AFM current maps (bottom) of perovskite films: CH_3_NH_3_PbI_3_ (a, f); CH_3_NH_3_PbI_3_ modified with 5 mmol L^–1^ BAI (b, g); CH_3_NH_3_PbI_3_ modified with 50 mmol L^–1^ BAI (c, h); CH_3_NH_3_PbI_3_ modified with 0.5 mmol L^–1^ BDAI_2_ (d, i); and CH_3_NH_3_PbI_3_ modified with 5 mmol L^–1^ BDAI_2_ (e, j). All topography (top) and c-AFM (bottom) images share the same color scales, displayed on the right side of the figures.

Pristine 3D perovskite films (Figure 6a,f) exhibited heterogeneous current distribution with several nonconductive domains, consistent with the existence of charge trapping states at grain boundaries. Using the perovskite modified with a 5 mmol L^–1^ BAI solution (Figure 7b,g), an improvement in conductivity uniformity is observed, suggesting effective passivation of surface defects. In contrast, films modified with a 50 mmol L^–1^ BAI solution (Figure 6c,h) showed a pronounced decrease in local current, possibly due to the formation of thick, insulating 2D RP layers. For DJ-type perovskite prepared using 0.5 mmol L^–1^ BDAI_2_ solution (Figure 6d,i), the highest current response is observed, indicating optimal passivation without disrupting charge transport. However, using BDAI_2_ 5 mmol L^–1^ solution (Figure 6e,j) led to a reduced conductivity, though to a lesser extent than BAI, suggesting that the DJ phase can better tolerate increased spacer loading. These c-AFM findings reinforce the role of spacer concentration in modulating interfacial conductivity, setting the stage for further analysis of device-level performance.

**Figure 7:**
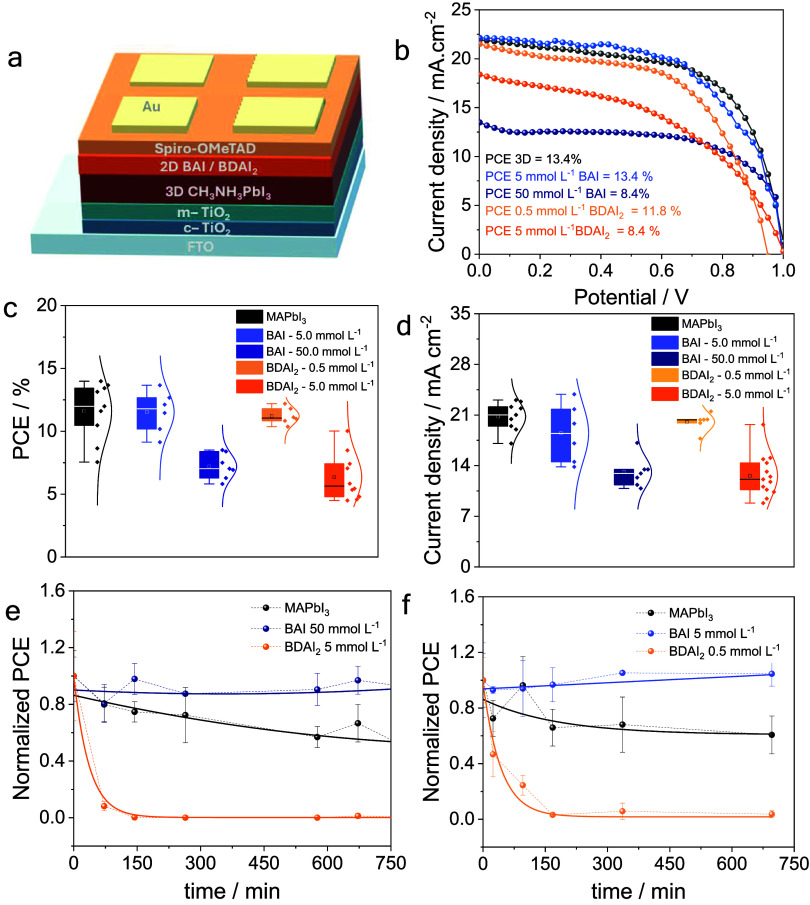
Device architecture of PSCs incorporating 2D|3D perovskites (a), best *JV* curves determined for different compositions of the 2D layer (b), box plots of power conversion efficiency (PCE) (c), and current density (d) for the as-prepared devices. Durability assessment based on normalized PCE of PSCs prepared using [BAI] = 50 mmol L^–1^ or [BDAI_2_] = 5 mmol L^–1^ (e) and [BAI] = 5 mmol L^–1^ or [BDAI_2_] = 0.5 mmol L^–1^ (f) spacer concentrations, using ISOS-D1 protocol for 3 different solar cells. Dashed lines connect the experimental data points, and solid lines represent the overall degradation trend.

Photovoltaic performances of PSCs based on 3D or 2D|3D perovskite films were evaluated using *JV* curves (Figure 7) and are summarized in Table S1. The devices were fabricated by stacking films onto FTO surface, resulting in the structure FTO|c-TiO_2_|m-TiO_2_|3D-perovskite|2D-perovskite|spiro-OMeTAD|Au, as illustrated in Figure 7a. The current density–voltage (*JV*) curves for the best-performing devices, measured in the forward direction under 1 sun (AM 1.5G; Figure 7b), reveal that increasing the concentration of the 2D spacer solution diminishes the photovoltaic performance and the power conversion efficiency (PCE).

It is also important to note here that all devices were fully fabricated in ambient air (40–50% RH), including solution preparation, perovskite deposition and HTL processing, without glovebox steps or encapsulation. Under these conditions, CH_3_NH_3_PbI_3_-based architectures typically exhibit lower PCE compared with inert-atmosphere processed devices, due to increased defect density, reduced crystallinity and more challenging control of the perovskite|HTL interface.^59^ Therefore, the efficiencies obtained here fall within the range commonly reported for fully air-processed CH_3_NH_3_PbI_3_ cells.^59–61^

Boxplots (Figure 7c) corroborate the trend observed for the Best JV curves, and it is possible to observe that devices prepared by using solutions with low concentrations of BAI (5 mmol L^–1^) or BDAI_2_ (0.5 mmol L^–1^) exhibit average PCE values comparable to those of pristine 3D perovskite. In contrast, higher concentrations (50 mmol L^–1^ BAI or 5 mmol L^–1^ BDAI_2_) lead to a notable efficiency drop. A similar effect is observed for the short-circuit current density (Figure 7d), indicating that excessive spacer incorporation at the perovskite|HTL interface increases the electrical resistance due to the insulating nature of the 2D phase, thereby hindering charge-carrier transport across the interface, being the main responsible for low PCE values.

The open-circuit voltage (*V*_OC_) values remained in a narrow range (0.85–0.94 V) for all compositions (Table S1 and Figure S10), suggesting that the incorporation of alkylammonium spacers does not noticeably affect the interfacial energetics or the built-in potential of the devices. In contrast, the fill factor (FF) exhibits two distinct behaviors: for BAI, FF decreases from 0.62 to 0.53 when the concentration is increased from 5 to 50 mmol L^–1^; for both concentrations of BDAI_2_, the fill factor (FF) remains slightly lower than in the reference and BAI-based devices. This behavior can be attributed to the intrinsic rigidity of the DJ-type spacer, which promotes stronger ionic bonding and limited structural flexibility, potentially generating interfacial strain or small inhomogeneities that hinder charge extraction.^62^ Additionally, the divalent nature of BDAI_2_ may lead to less uniform coverage, as also evidenced by the c-AFM (Figure 6i). These factors contribute to a lower FF even at low BDAI_2_ concentrations, although the overall efficiency remains comparable to the pristine device at 0.5 mmol L^–1^

The external quantum efficiency (EQE) spectra of representative devices (Figure S11a,b) exhibit trends consistent with their *JV* characteristics. The integrated photocurrent densities (Figure S11c,d) derived from the EQE curves are in reasonable agreement with the measured short-circuit current densities (Table S1), confirming the relation between optical and electrical responses. Devices with low spacer concentrations (BAI 5 mmol L^–1^ and BDAI_2_ 0.5 mmol L^–1^) display broader and more intense photoresponse, while higher spacer loadings (BAI 50 mmol L^–1^ and BDAI_2_ 5 mmol L^–1^) show attenuated signals, consistent with reduced charge extraction through more insulating 2D-rich interfacial regions.

Durability was assessed through the ISOS-D1 protocol over 700 min for PSCs using cationic spacers deposited from solutions of different concentrations (Figure 7e,f). Comparing the performance behavior of the samples, it is evident that the DJ-type spacer (BDAI_2_) exhibited the poorest stability, regardless of spacer concentration. The structural instability was so severe that this modification resulted in lower durability than that of CH_3_NH_3_PbI_3_.

The evolution of *J*_SC_, FF, and *V*_OC_ (Figure S12) supports these findings. Devices with the highest BDAI_2_ loading (5 mmol L^–1^) exhibit a rapid drop in all parameters, consistent with the development of resistive and poorly connected domains that hinder charge extraction and accelerate degradation. In contrast, BAI-based cells display remarkable stability, with negligible changes in *J*_SC_, *V*_OC_, and FF throughout the test, reflecting the formation of defect-tolerant RP-type interfaces. For the low-concentration BDAI_2_ devices (0.5 mmol L^–1^), the degradation primarily affects *J*_*SC*_, while FF decreases more gradually, indicating that the diode remains functional but limited by reduced carrier collection.^63^

The accelerated degradation of BDAI_2_ can be a result of the interplay between humidity, lattice rigidity, and interfacial disorder. BDAI_2_ is hygroscopic and strongly bound to the inorganic framework, as already discussed.^64,65^ Under the ambient fabrication conditions used here (40–50% RH), this rigidity may promote strain accumulation, disrupting uniform layer formation, consistent with reports that processing conditions accentuate disorder during low-*n* 2D crystallization.^51^ These heterogeneous domains are more susceptible to moisture-assisted defect formation,^65^ and once operational stress is applied, these strained and partially disordered regions further facilitate cation migration and interfacial degradation, ultimately leading to rapid performance loss.^55^ Thus, the degradation originates from the coupled effects of moisture, lattice strain, and processability of the DJ interface—rather than from any single factor acting independently.

In contrast, perovskite modification using RP-type spacers (Figure 7e,f) resulted in improved stability, maintaining the initial PCE values for both tested concentrations, while the efficiency of pristine CH_3_NH_3_PbI_3_ decreased over time. Therefore, even if the RP spacer interacts as a passivator, it still enhances overall durability.

To further investigate potential cation migration, UV–vis spectra were collected from the devices over time (Figure S13). Notably, the 2D-related absorption band in BAI (50 mmol L^–1^) decreased within a few days, whereas perovskite films modified with BDAI_2_ (5 mmol L^–1^) underwent significant degradation. Since this effect was observed only in operational devices and not in standalone films, it reinforces the idea that operational stress accelerates the degradation process of BDAI_2_ samples.^55,66^

Electrochemical Impedance Spectroscopy (EIS) was employed to investigate electronic and electrochemical interfacial processes in PSCs at open-circuit potential (*V*_OC_), under 1-sun illumination (Figure 8). The Nyquist plots typically display an arc at high frequencies (HF > 100 kHz), which reflects the response of fast interfacial processes related to the charge carriers^67,68^ and they were fitted by the linear Voight equivalent circuit (inset Figure 8), consisting of a series resistance (R1) and an R/C circuit (R2 and CPE1) representing the HF region. In this circuit, a Constant Phase Element (CPE) accounts for nonideal capacitance behavior often observed at real PSC interfaces and is used instead of an ideal capacitor. The HF region is commonly attributed to the resistive and capacitive properties at the interfaces between the charge transport layers and the perovskite.^69,70^ The values determined for R1, R2 and CPE1 are in Table 1.

**Figure 8:**
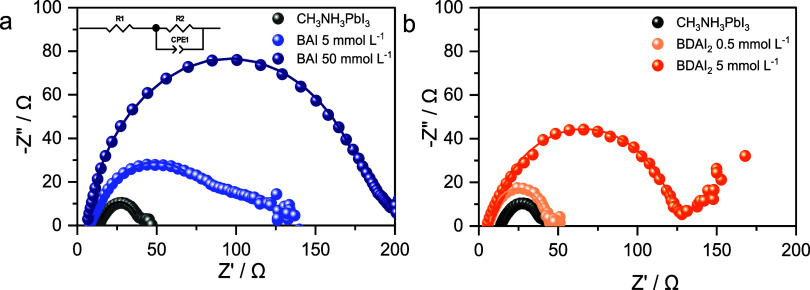
Nyquist diagrams of PSCs modified with BAI (a) and BDA*I*_2_(*b*) (Experimental (●) and fitted (―) data; Frequency range = 1 MHz–0,1 Hz at *V*_OC_; *V*_AC_ = 10 mV; *P*_irr_ = 1 sun). The *x*-axis corresponds to the real part of the impedance (*Z*′), while the *y*-axis shows the negative imaginary part (−*Z*″). Inset: Voight equivalent circuit used in fitting.

**Table 1:**
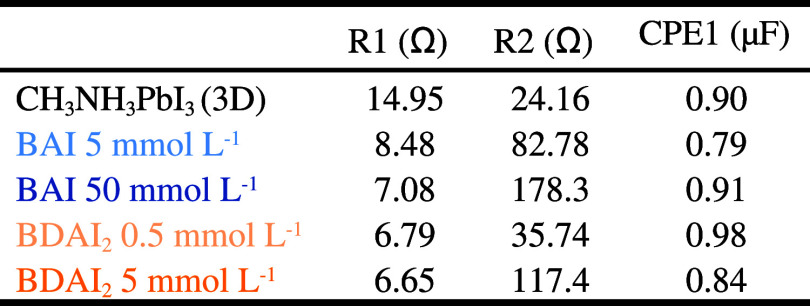
Electrical Resistances (R1 and R2) and Constant Phase Element (CPE1), Determined by Fitting the HF Region of Nyquist Diagrams for Perovskite Solar Cells Using 3D Perovskite or Modified by Using Solutions of BAI or BDAI_2_ in Different Concentrations^a^

a (Frequency range = 1 MHz – 0,1 Hz at *V*_OC_; *V*_AC_ = 10 mV; *P*_irr_ = 1 sun).

In the proposed equivalent circuit, the series resistance (R1) is associated with external electric contacts, such as wires, connections and FTO substrates, and it can be observed that the values determined for all samples are similar. In contrast, the charge transfer resistance (R2), has an intrinsic relationship to the interfacial charge extraction efficiency between the perovskite|HTL|Au interfaces.^71^ The R2 values varied significantly across the samples, revealing a clear relationship with the amounts of the 2D spacer cations used, leading to either passivation of 3D perovskite or the formation of 2D|3D heterostructures.

Among the samples investigated, 3D CH_3_NH_3_PbI_3_ showed the lowest R2 value, indicating efficient charge-carrier transport across the interfaces.^72^ R2 values determined for devices treated with 2D spacers are higher than those determined for pristine 3D devices. This is a consequence of the presence of spacers that introduce electrical resistance to charge carrier transport across the interfaces due to the insulator characteristic of the aliphatic chain. Higher spacer concentrations tend to yield more extensive 2D coverage, which may also lead to a thicker interfacial 2D region. This trend is reflected in the increase in *R*_2_ values, indicating that the degree (and possibly the thickness) of 2D formation influences interfacial charge extraction. Notably, samples prepared using [BAI] = 50 mmol L^–1^ exhibited the highest resistance (178.3 Ω) among all samples, suggesting that excessive incorporation of 2D material may introduce charge extraction barriers by the organic cations. In contrast, [BDAI_2_] = 0.5 mmol L^–1^ showed significantly lower resistance (35.74 Ω) compared with [BDAI_2_] = 5 mmol L^–1^ (117.4 Ω), indicating that an optimal amount of 2D spacer can be achieved, resulting in more efficient interfacial charge carrier transport across the interfaces, reducing their recombination.^72,73^

The Constant Phase Element (CPE1) represents a nonideal capacitance of perovskite|HTL|Au layers and showed similar values for all devices investigated. Considering that the high-frequency capacitance in PSCs is primarily governed by the geometric capacitance of the bulk 3D CH_3_NH_3_PbI_3_ perovskite layer,^70^ rather than the differences introduced by the surface modifications, the behavior observed is consistent with this model.

To provide a clearer overview of how spacer chemistry and concentration simultaneously affect photovoltaic performance, interfacial charge transport and device durability, we summarize in (Table 2) the key device parameters (*J*_sc_, FF, PCE), stability retention under the ISOS-D1 protocol, and charge-transfer resistance (*R*_2_) obtained from impedance spectroscopy for all compositions investigated. The data were extracted from Table S1 (photovoltaic parameters), Figure 7e–f (durability) and Table 1 (EIS results).

**Table 2:**
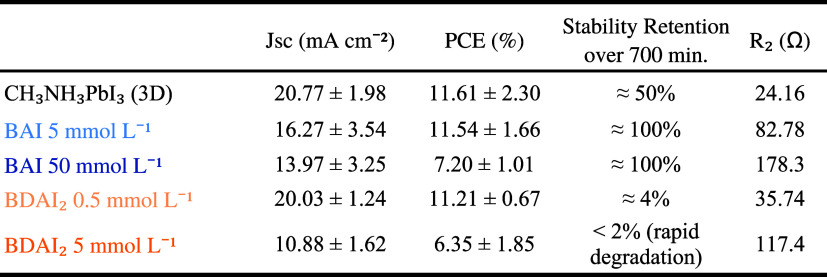
Comparative Overview of Short-Circuit Current Density (*J*_sc_), Photovoltaic Efficiency (PCE), Durability, and Interfacial Resistance (*R*_2_)

At low concentrations, both BAI (5 mmol L^–1^) and BDAI_2_ (0.5 mmol L^–1^) primarily act as surface passivators, preserving the 3D framework and yielding PCE values comparable to pristine CH_3_NH_3_PbI_3_. However, their long-term behavior diverges markedly. BAI forms a thin RP-like interfacial layer that improves environmental resistance without hindering charge extraction, resulting in nearly complete stability retention. In contrast, the hygroscopic nature of BDAI_2_ and the local inhomogeneity introduced during ambient processing limit durability, despite its favorable electronic properties and relatively low *R*_2_.

At higher concentrations, low-dimensional structure formation becomes dominant. For BAI (50 mmol L^–1^), RP layering increases *R*_2_ and lowers *J*_sc_ and PCE due to constrained charge transport, yet the resulting interfaces remain highly moisture-resistant and remain stable over time. BDAI_2_ at 5 mmol L^–1^ behaves differently: although it produces low-dimensional domains, the rigid diammonium spacer induces strain and disrupts uniform stacking under humid processing conditions, leading to rapid degradation and <20% retention despite *R*_2_ being lower than in the BAI analogue. This highlights that operational stability is governed not solely by interfacial transport but by the lattice adaptability and environmental robustness of the spacer–perovskite interaction.

Overall, these results indicate that optimizing 2D|3D perovskites requires balancing spacer concentration with the structural adaptability of the spacer layer and with controlled processing conditions. Low loadings of monoammonium BAI provide the most favorable compromise, offering defect passivation and moisture resistance while maintaining efficient charge extraction. In contrast, DJ-type spacers such as BDAI_2_ are more susceptible to ambient processing and tend to introduce strain-related defects that accelerate degradation.

## Conclusions

This study demonstrates that the chemistry and concentration of alkylammonium spacers critically govern the balance between stability and efficiency in 2D|3D perovskite solar cells. The contrasting behavior of BAI and BDAI_2_ shows that structural rigidity alone does not ensure durability; BDAI_2_ unexpectedly accelerated degradation under ambient conditions, whereas BAI promoted dynamic phase evolution that suppressed failure pathways and prolonged operational device lifetime. These results show that spacer molecules act not only as structural modifiers but also as active regulators of charge transport and device durability.

By clarifying these mechanisms, our work provides practical insights into spacer selection and concentration in hybrid perovskites, advancing strategies for achieving stable and more durable devices. Overall, the findings underline the importance of molecular engineering strategies for reconciling efficiency with long-term durability, a central requirement for commercialization. Future research should explore mixed-spacer systems, functionalized cations, and controlled ambient-processing routes to further optimize the interplay among charge dynamics, phase stability, and resistance to degradation.

## Supplementary Material



## Data Availability

Experimental data supporting this study, including UV–vis spectroscopy, *JV* curves, XRD, and PL spectra, are available in the Zenodo repository at 10.5281/zenodo.17257899. Additional details are provided in the Supporting Information.

## Notes

Additional scanning electron microscopy images of pristine and spacer-modified perovskite films (Figures S1–S6), followed by UV–vis absorption spectra, including differential and time-dependent measurements of the films (Figures S7–S9); statistical photovoltaic parameters are summarized in Table S1, while additional device statistics (Figure S10), external quantum efficiency spectra and integrated photocurrent analyses (Figure S11), and the evolution of device stability parameters (*J*_SC_, FF and *V*_OC_) under ISOS-D1 conditions (Figure S12) are provided for the corresponding solar cells; UV–vis spectra collected from complete devices during aging are shown in Figure S13 (PDF)
