# Supplementary material for: Alkylammonium Spacer-Directed Charge-Transfer in 2D|3D Perovskite Solar Cells
Source: ACS Omega. 2026 Jan 28;11(5):8061–73. doi: 10.1021/acsomega.5c10453 (PMC12902966; doi:10.1021/acsomega.5c10453)
Supplement: Supplementary file 1 [file ao5c10453_si_001.pdf]

Support Information

Alkylammonium Spacer-Directed Charge-Transfer in 2D|3D Perovskite Solar Cells

Barbara Scola Rodrigues [a], Lucas Polimante [a], Cleyton Alexandre Biffe [b], Carlos Alberto Rodrigues Costa [b], André Sarto Polo [a]*

[a] Centro de Ciências Naturais e Humanas, Universidade Federal do ABC, 09210-580, Santo

André, SP, Brazil.

[b] Brazilian Nanotechnology National Laboratory (LNNano), Brazilian Center for Research in Energy and Materials (CNPEM), Zip Code 13083-970, Campinas, Sao Paulo, Brazil.

*andre.polo@ufabc.edu.br

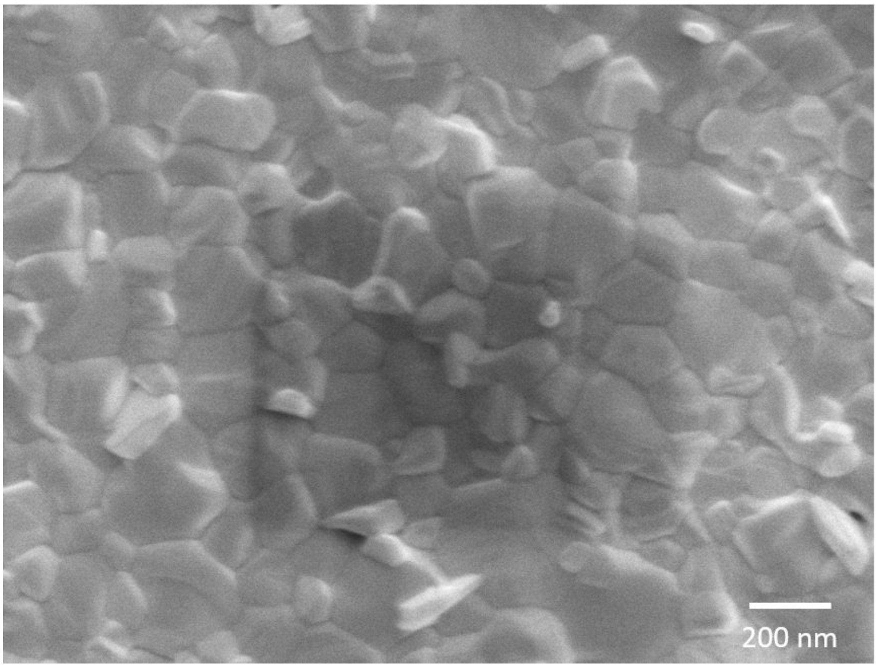

Figure S 1. $CH_3NH_3PbI_3$ perovskite surface after deposition of 100 μLof isopropyl alcohol

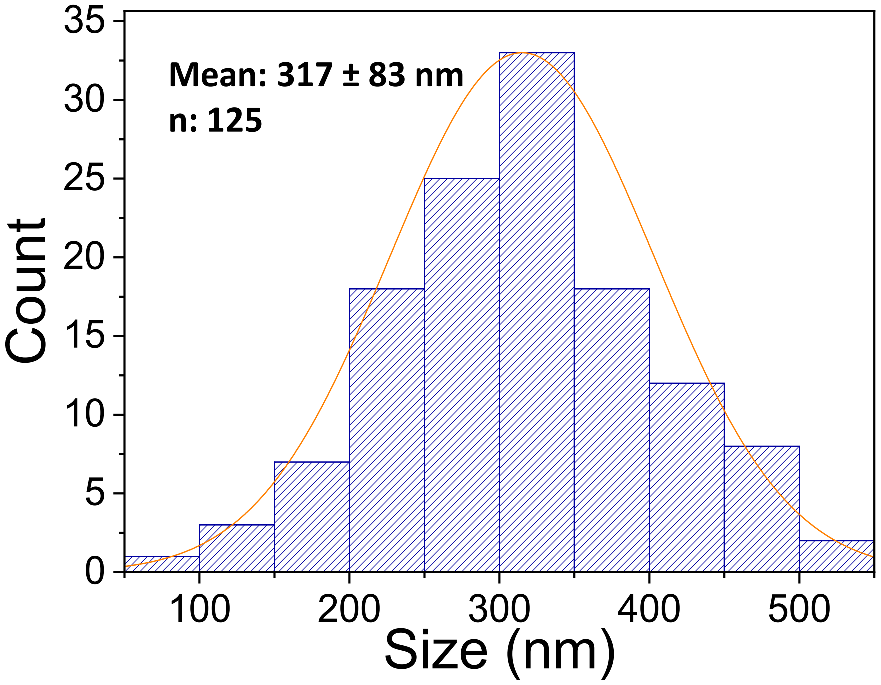

Figure S 2. Histogram of particle size measured for $CH_3NH_3PbI_3$

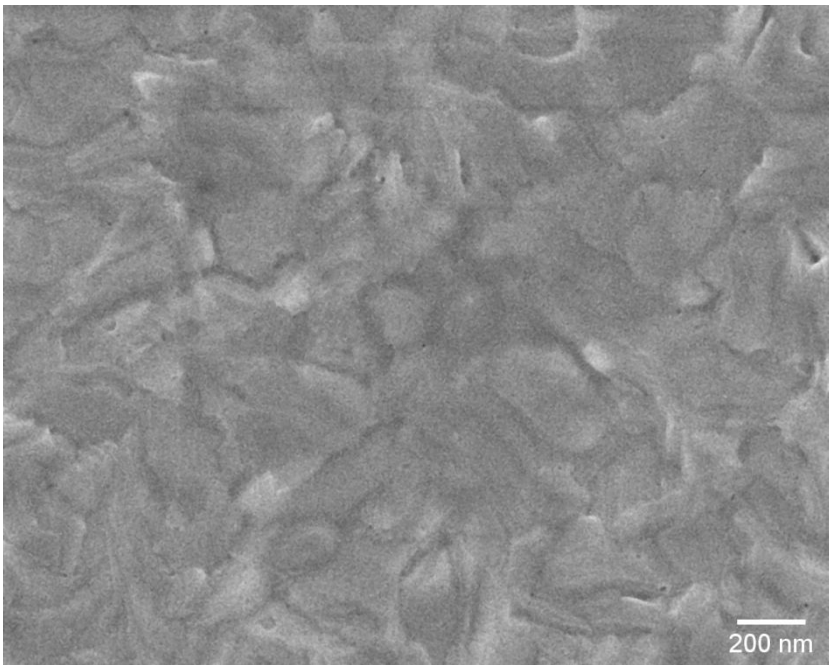

Figure S 3. $CH_3NH_3PbI_3$ perovskite modified with 50 mmol L^{-1} BAI.

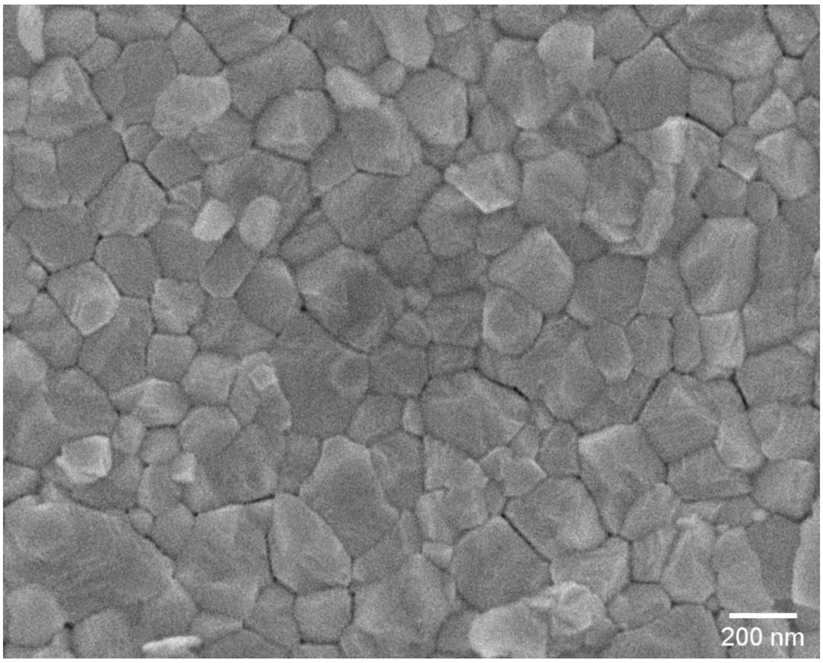

Figure S 4. $CH_3NH_3PbI_3$ perovskite modified with 5 mmol L^{-1} BAI.

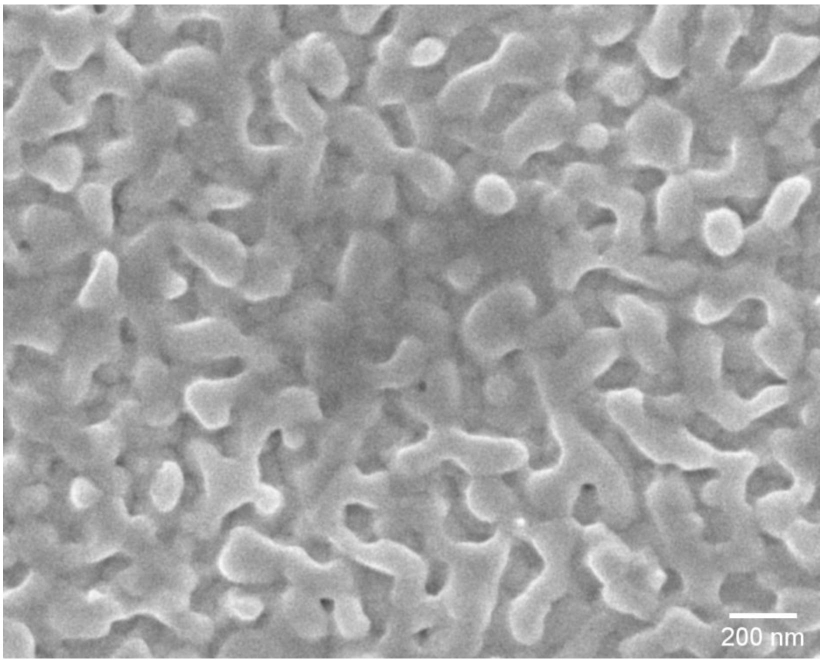

Figure S 5. $CH_3NH_3PbI_3$ perovskite modified with 5 mmol L^{-1} $BDAI_2$.

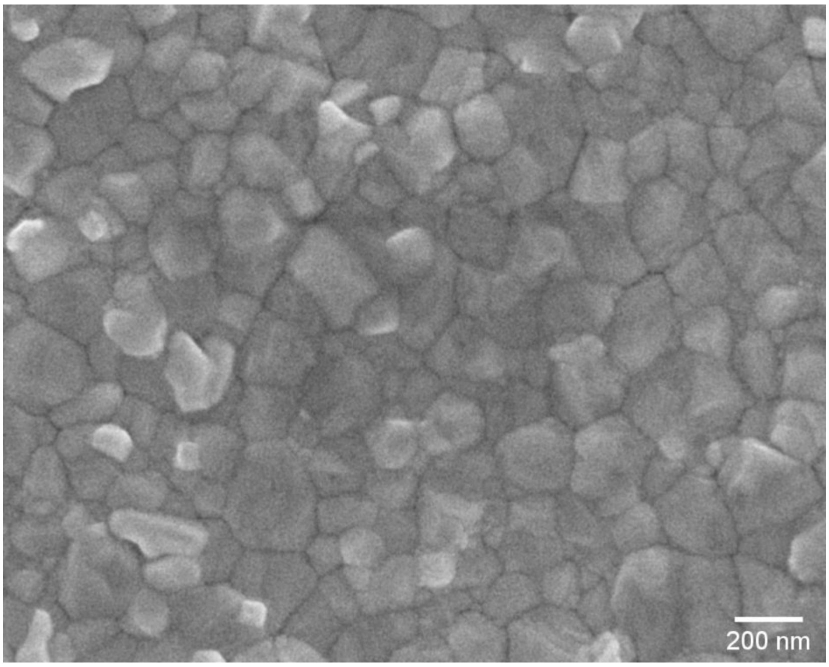

Figure S 6. $CH_3NH_3PbI_3$ perovskite modified with 0.5 mmol L^{-1} $BDAI_2$.

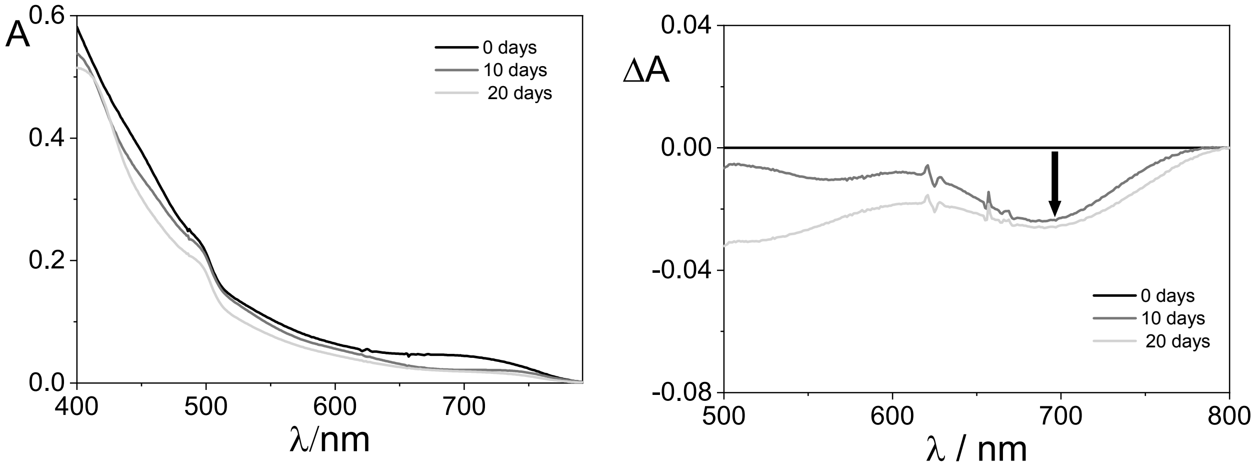

Figure S 7. UV-vis spectra with $CH_3NH_3PbI_3$ perovskite, over the days (left) and differential absorption (right).

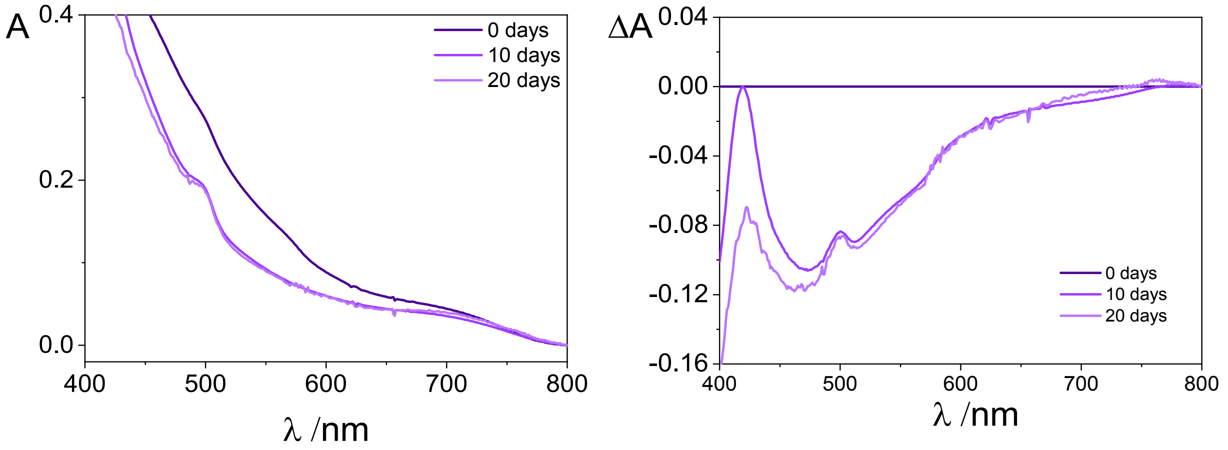

Figure S 8. UV-vis spectra of 2D│3D synthetized with 5 mmol L^{-1} BAI over the days (left) and differential absorption (right).

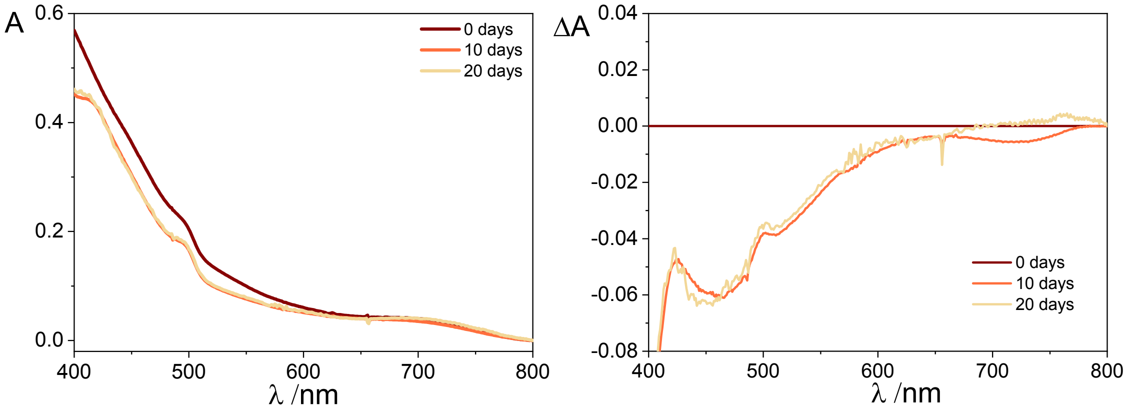

Figure S 9. UV-vis spectra of 2D│3D synthetized with 0.5 mmol L^{-1} $BDAI_2$ over the days (left) and differential absorption (right).

Table S 1. Average photovoltaic parameters for perovskite solar cells incorporating different 2D spacer cations. PCE: power conversion efficiency; V_{OC}: open-circuit voltage; J_{SC}: short-circuit current density; FF: fill factor

Device	Jsc / mA cm^{-2}	Voc / V	FF / %	PCE / %
$CH_3NH_3PbI_3$	20.77 ± 1.98	0.85 ± 0.13	64.97 ± 7.22	11.61 ± 2.30
BAI 5 mmol L^{-1}	16.27 ± 3.54	0.90 ± 0.12	64.95 ± 2.27	11.54 ± 1.66
BAI 50 mmol L^{-1}	13.97 ± 3.25	0.94 ± 0.13	46.11 ± 9.14	7.20 ± 1.01
$BDAI_2$ 0.5 mmol L^{-1}	20.03 ± 1.24	0.86 ± 0.10	57.02 ± 2.82	11.21 ± 0.67
$BDAI_2$ 5 mmol L^{-1}	10.88 ± 1.62	0.85 ± 0.07	45.63 ± 13.06	6.35 ± 1.85

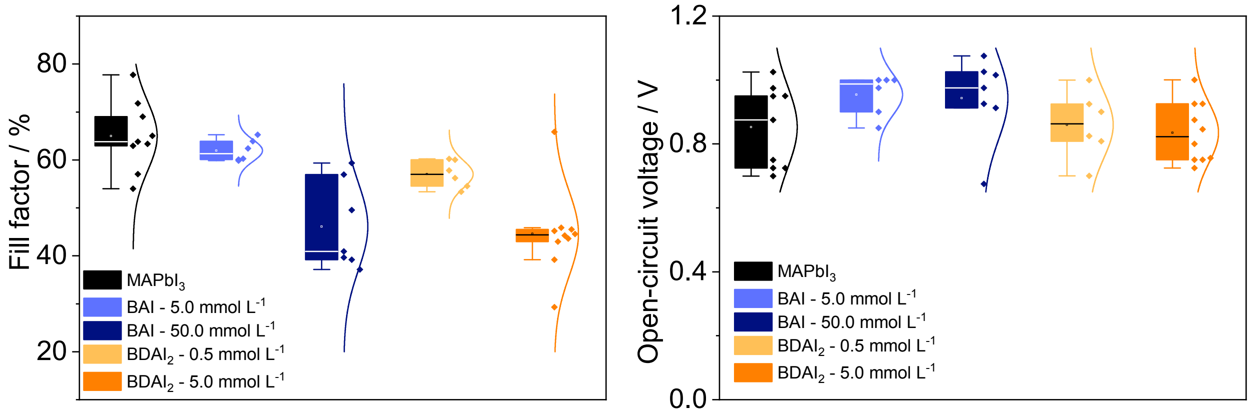

Figure S 10. Boxplots of (a) open-circuit voltage (V_{OC}) and (b) fill factor (FF) for perovskite solar cells prepared with different 2D spacer concentrations.

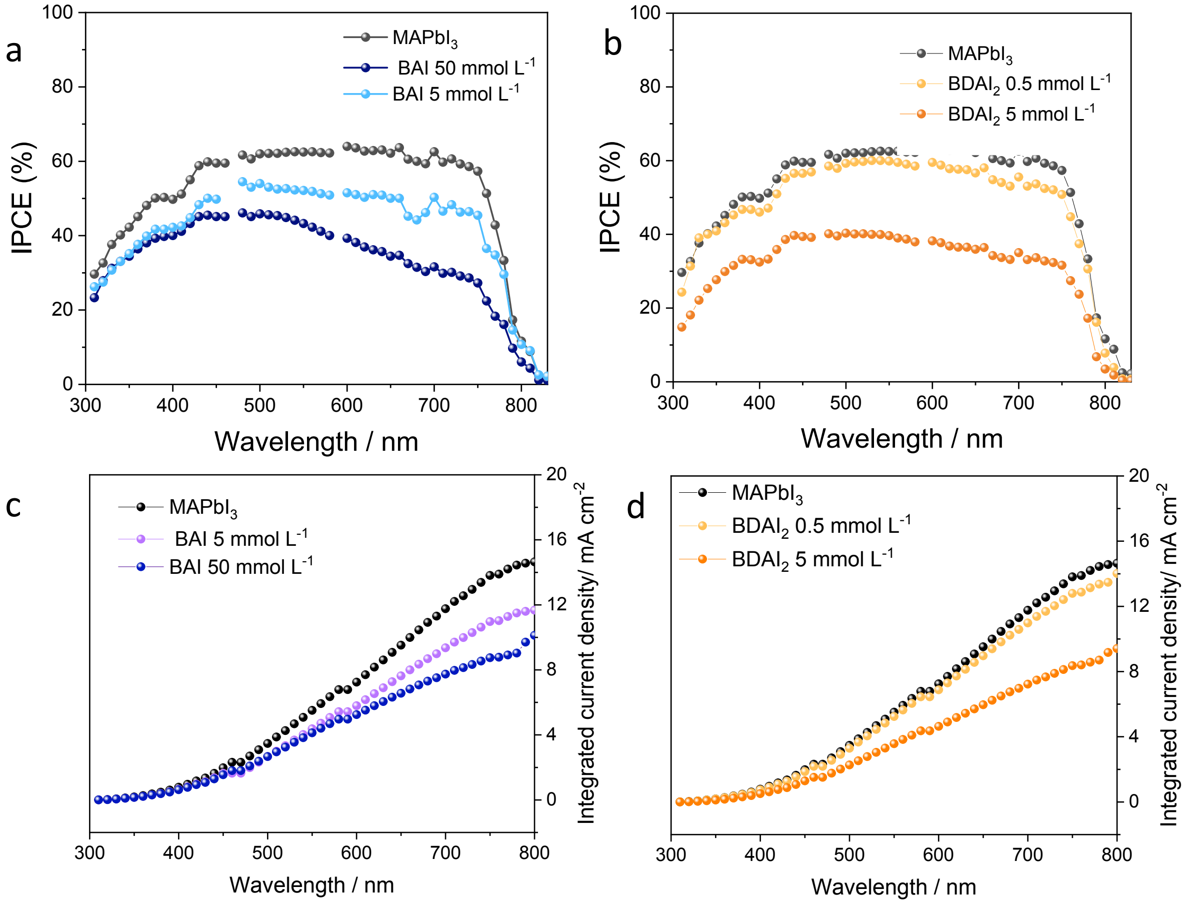

Figure S 11. External quantum efficiency (EQE) spectra (top) and corresponding integrated photocurrent densities (bottom) for perovskite solar cells prepared with different 2D spacer concentrations. (a, c) Devices modified with butylammonium iodide (BAI) and (b, d) with butyl-1,4-diammonium diiodide ($BDAI_2$), compared to pristine $CH_3NH_3PbI_3$.

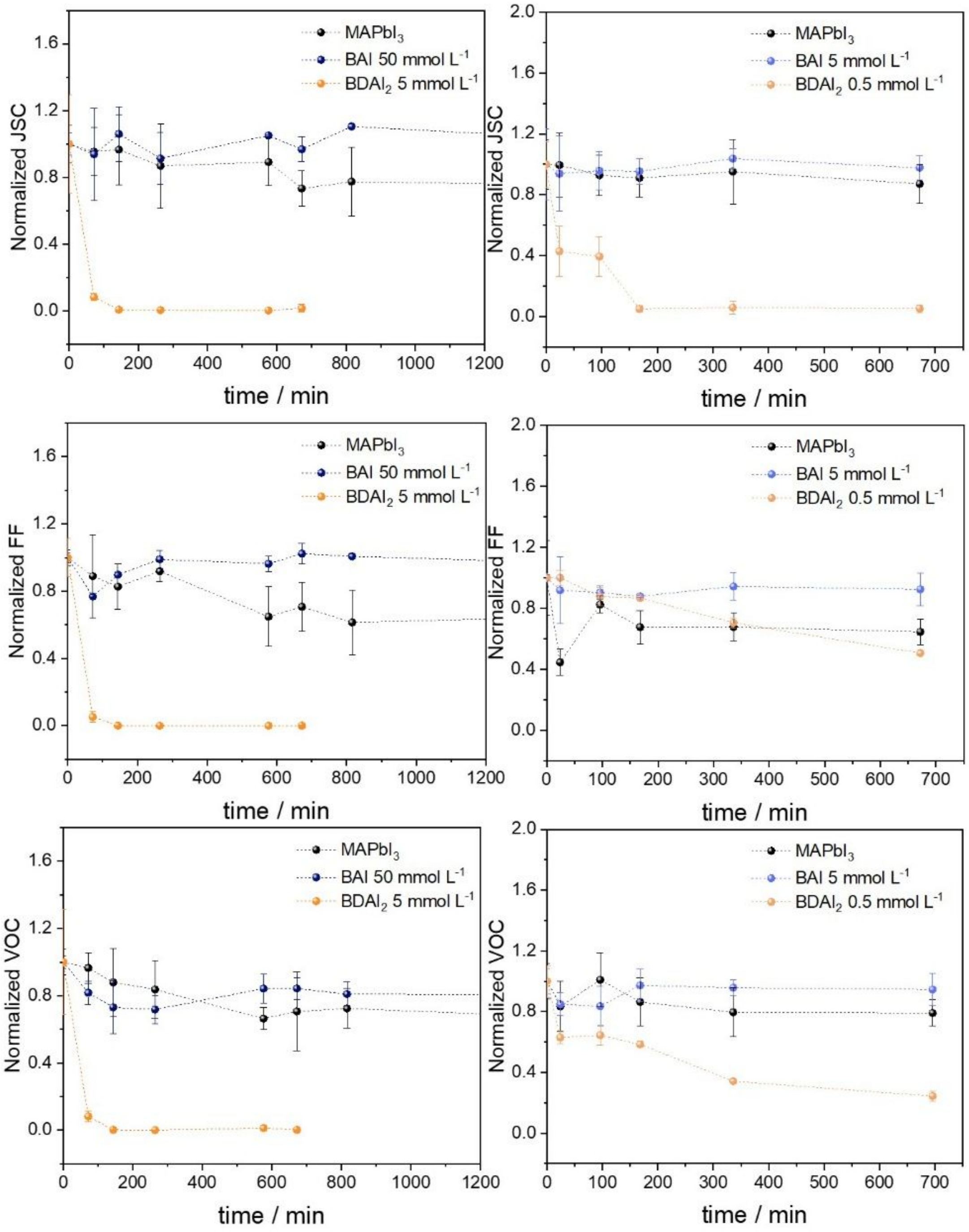

Figure S 12. Normalized evolution of the short-circuit current density (J_{SC}), fill factor (FF), and open-circuit voltage (V_{OC}) for perovskite solar cells measured under ISOS-D1 protocol. The left panels compare devices containing high spacer concentrations—BAI 50 mmol L^{-1} and $BDAI_2$ 5 mmol L^{-1}—while the right panels correspond to lower concentrations—BAI 5 mmol L^{-1} and $BDAI_2$ 0.5 mmol L^{-1}—relative to pristine $CH_3NH_3PbI_3$. Error bars represent the standard deviation from 3 devices.

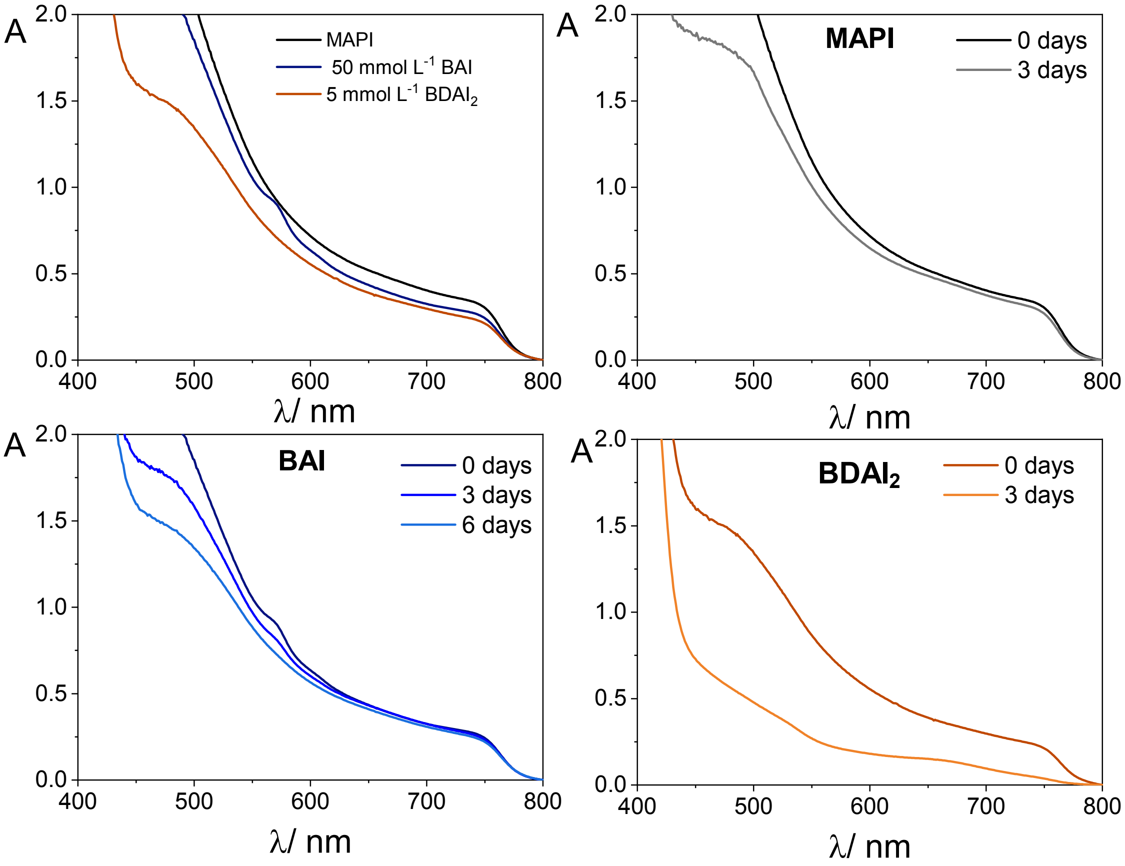

Figure S 13. UV-vis spectra of devices based on $CH_3NH_3PbI_3$ perovskite, 2D│3D perovskites obtained with 50 mmol L-1 of BAI and 5 mmol L-1 $BDAI_2$
